# Supplementary material for: Integrative Proteomic and Phosphoproteomic Analyses of Hypoxia-Treated Pulmonary Artery Smooth Muscle Cells
Source: Proteomes. 2022 Jun 28;10(3):23. doi: 10.3390/proteomes10030023 (PMC9326561; doi:10.3390/proteomes10030023)
Supplement: Supplementary file 1 [file proteomes-10-00023-s001.zip › Supplementary figures.pdf]

Supplementary Figures

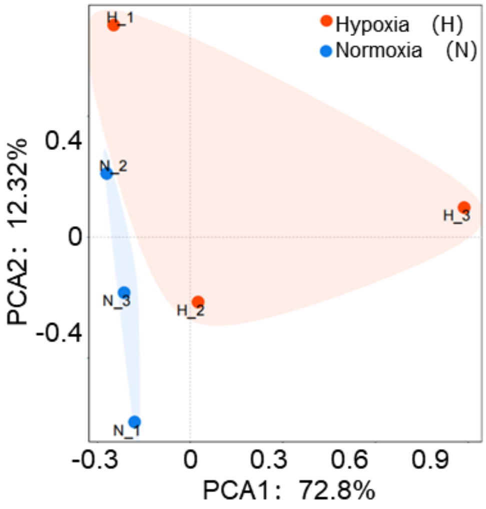

Figure S1. Principal component analysis (PCA) of the protein abundances in proteome study.

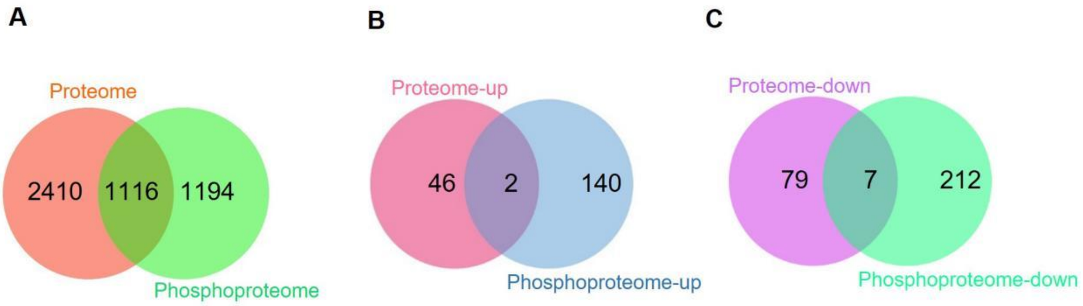

Figure S2. Venn diagram showing proteins and phosphoproteins in proteome and phosphoproteome analysis. (A) Total proteins and phosphoproteins. (B) Upregulated proteins and upregulated phosphoproteins. (C) Downregulated proteins and downregulated phosphoproteins.

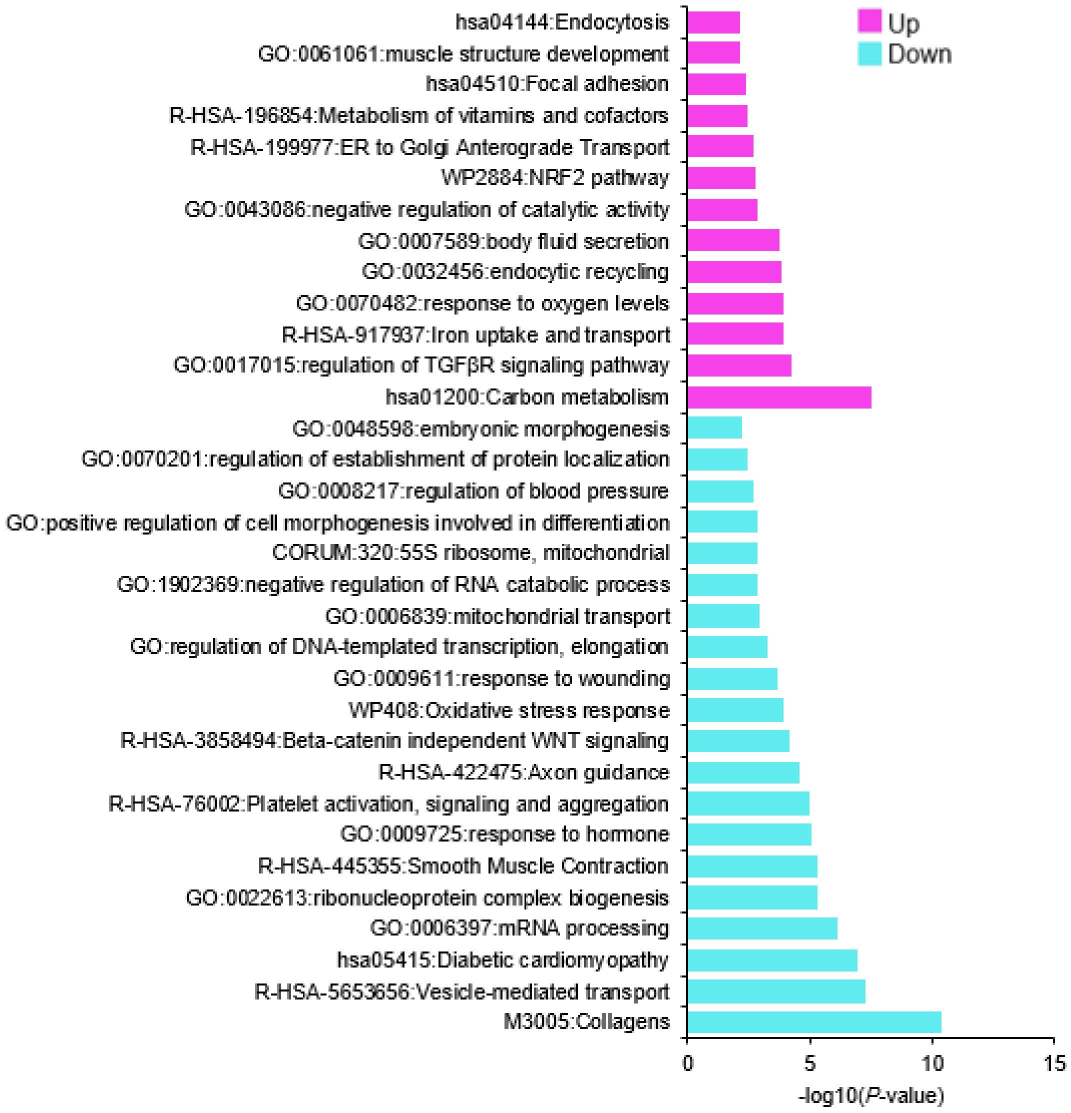

Figure S3. Comprehensive Pathway enrichment analysis of significantly changed proteins (P < 0.05, ∣log2 (fold change) ∣ > log2 (1.1)) in hypoxia-treated cells compared with the control cells (normoxia).

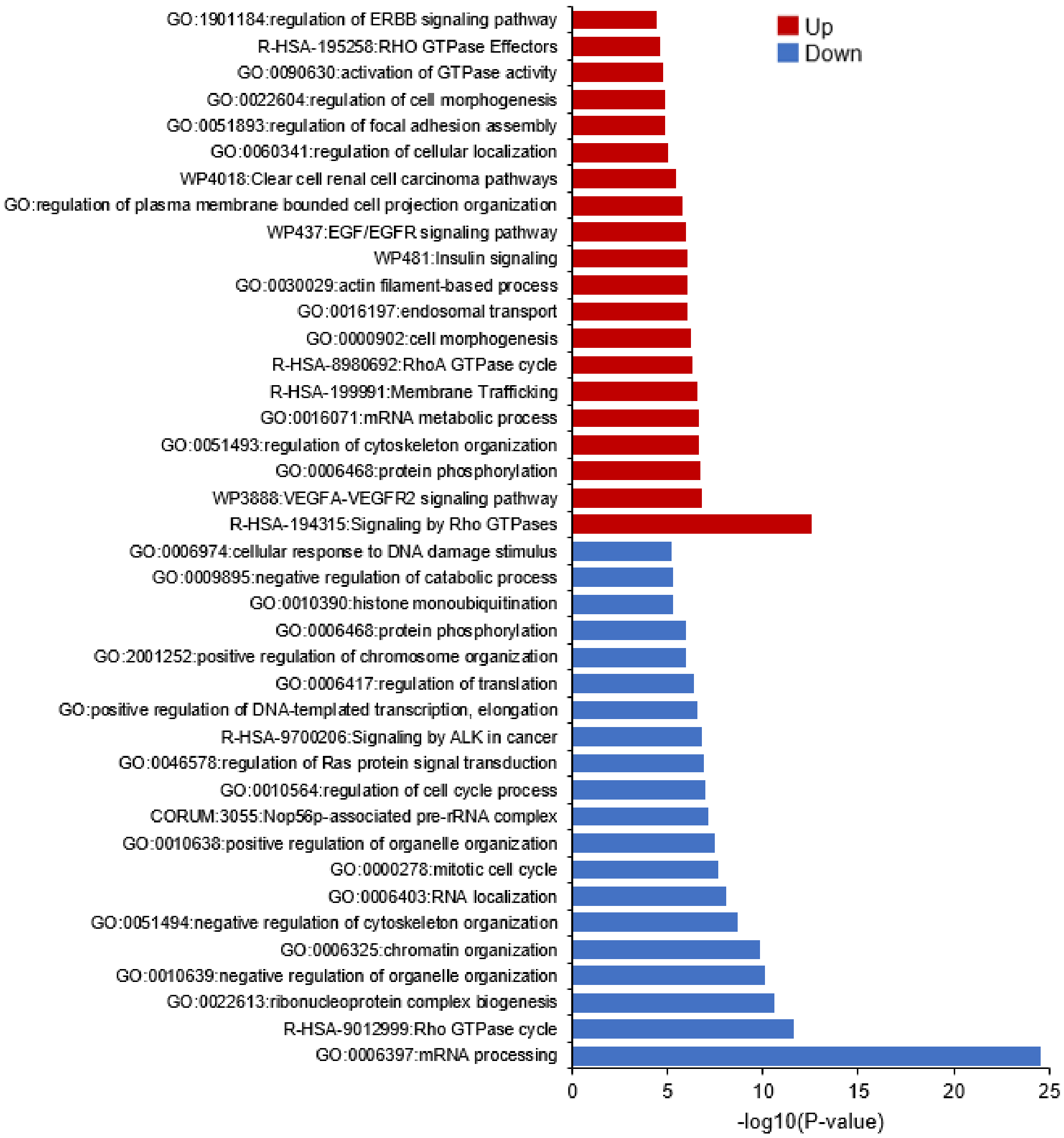

Figure S4. Comprehensive Pathway enrichment analysis of significantly changed phosphoproteins ($P < 0.05$, $|\log2$ (fold change)$| > \log2$ (1.1)) in hypoxia-treated cells compared with the control cells (normoxia).
