# Supplementary material for: Integrative Proteomic and Phosphoproteomic Analyses of Hypoxia-Treated Pulmonary Artery Smooth Muscle Cells
Source: Proteomes. 2022 Jun 28;10(3):23. doi: 10.3390/proteomes10030023 (PMC9326561; doi:10.3390/proteomes10030023)
Supplement: Supplementary file 1 [file proteomes-10-00023-s001.zip › Table S2.pdf]

Table S2

Proteins significantly changed in proteome analysis of hypoxia-treated hPASMCs

**UniProtKB ID**	**Gene name**	**Protein name**	**Fold change**
Q15904	*ATP6AP1*	V-type proton ATPase subunit S1	1.62
Q9BY76	*ANGPTL4*	Angiopoietin-related protein 4	1.44
O43570	*CA12*	Carbonic anhydrase 12	1.44
O00743	*PPP6C*	Serine/threonine-protein phosphatase 6 catalytic subunit	1.42
Q8NB37	*GATD1*	Glutamine amidotransferase-like class 1 domain-containing protein 1	1.42
Q9UJJ9	*GNPTG*	N-acetylglucosamine-1-phosphotransferase subunit gamma	1.39
Q7Z5L9	*IRF2BP2*	Interferon regulatory factor 2-binding protein 2	1.32
A6NFQ2	*TCAF2*	TRPM8 channel-associated factor 2	1.32
P02794	*FTH1*	Ferritin heavy chain	1.30
Q15796	*SMAD2*	Mothers against decapentaplegic homolog 2	1.29
Q9UJW0	*DCTN4*	Dynactin subunit 4	1.29
O00506	*STK25*	Serine/threonine-protein kinase 25	1.25
P05155	*SERPING1*	Plasma protease C1 inhibitor	1.24
Q9NR46	*SH3GLB2*	Endophilin-B2	1.23
Q9H9E3	*COG4*	Conserved oligomeric Golgi complex subunit 4	1.23
P12277	*CKB*	Creatine kinase B-type	1.22
O43294	*TGFB1I1*	Transforming growth factor beta-1-induced transcript 1 protein	1.22
Q9Y4P3	*TBL2*	Transducin beta-like protein 2	1.18
P09104	*ENO2*	Gamma-enolase	1.18
Q6NUM9	*RETSAT*	All-trans-retinol 13,14-reductase	1.18
Q96JY6	*PDLIM2*	PDZ and LIM domain protein 2	1.18
O15530	*PDPK1*	3-phosphoinositide-dependent protein kinase 1	1.17
P11166	*SLC2A1*	Solute carrier family 2, facilitated glucose transporter member 1	1.16
Q96BP3	*PPWD1*	Peptidylprolyl isomerase domain and WD repeat-containing protein 1	1.16
Q06033	*ITIH3*	Inter-alpha-trypsin inhibitor heavy chain H3	1.15
Q3LXA3	*TKFC*	Triokinase/FMN cyclase	1.15
Q96L34	*MARK4*	MAP/microtubule affinity-regulating kinase 4	1.15
P11498	*PC*	Pyruvate carboxylase, mitochondrial	1.14
Q92615	*LARP4B*	La-related protein 4B	1.14
P49593	*PPM1F*	Protein phosphatase 1F	1.14
Q9NVS2	*MRPS18A*	39S ribosomal protein S18a, mitochondrial	1.14
P17813	*ENG*	Endoglin	1.13
P09341	*CXCL1*	Growth-regulated alpha protein	1.13

P21399	*ACO1*	Cytoplasmic aconitate hydratase	1.13
O75368	*SH3BGRL*	SH3 domain-binding glutamic acid-rich-like protein	1.13
Q9UBY9	*HSPB7*	Heat shock protein beta-7	1.12
P48163	*ME1*	NADP-dependent malic enzyme	1.12
P14384	*CPM*	Carboxypeptidase M	1.12
P17342	*NPR3*	Atrial natriuretic peptide receptor 3	1.12
Q9NZN4	*EHD2*	EH domain-containing protein 2	1.12
Q07960	*ARHGAP1*	Rho GTPase-activating protein 1	1.12
P08572	*COL4A2*	Collagen alpha-2	1.12
Q8IY33	*MICALL2*	MICAL-like protein 2	1.12
Q86TI2	*DPP9*	Dipeptidyl peptidase 9	1.12
P52789	*HK2*	Hexokinase-2	1.12
P07741	*APRT*	Adenine phosphoribosyltransferase	1.11
P24844	*MYL9*	Myosin regulatory light polypeptide 9	1.11
Q9P2B2	*PTGFRN*	Prostaglandin F2 receptor negative regulator	1.11
Q9GZU8	*PSME3IP1*	PSME3-interacting protein	0.91
Q9BVG4	*PBDC1*	Protein PBDC1	0.91
P05166	*PCCB*	Propionyl-CoA carboxylase beta chain, mitochondrial	0.91
O94826	*TOMM70*	Mitochondrial import receptor subunit TOM70	0.91
Q9NPJ3	*ACOT13*	Acyl-coenzyme A thioesterase 13	0.91
Q12849	*GRSF1*	G-rich sequence factor 1	0.91
Q13573	*SNW1*	SNW domain-containing protein 1	0.91
Q15758	*SLC1A5*	Neutral amino acid transporter B	0.91
Q96H20	*SNF8*	Vacuolar-sorting protein SNF8	0.90
P02786	*TFRC*	Transferrin receptor protein 1	0.90
P22626	*HNRNPA2B1*	Heterogeneous nuclear ribonucleoproteins A2/B1	0.90
O43242	*PSMD3*	26S proteasome non-ATPase regulatory subunit 3	0.90
P56537	*EIF6*	Eukaryotic translation initiation factor 6	0.90
Q9Y5B9	*SUPT16H*	FACT complex subunit SPT16	0.90
Q9NRF8	*CTPS2*	CTP synthase 2	0.90
Q96KR1	*ZFR*	Zinc finger RNA-binding protein	0.90
Q7Z417	*NUFIP2*	Nuclear fragile X mental retardation-interacting protein 2	0.89
Q9BXK5	*BCL2L13*	Bcl-2-like protein 13	0.89
Q12907	*LMAN2*	Vesicular integral-membrane protein VIP36	0.89
O60832	*DKC1*	H/ACA ribonucleoprotein complex subunit DKC1	0.89
E9PAV3	*NACA*	Nascent polypeptide-associated complex subunit alpha, muscle-specific form	0.89
P12109	*COL6A1*	Collagen alpha-1	0.89
Q14839	*CHD4*	Chromodomain-helicase-DNA-binding protein 4	0.89
P30050	*RPL12*	60S ribosomal protein L12	0.89
P67809	*YBX1*	Y-box-binding protein 1	0.89
Q9BUQ8	*DDX23*	Probable ATP-dependent RNA helicase DDX23	0.89

P04179	*SOD2*	Superoxide dismutase [Mn], mitochondrial	0.88
Q96EL3	*MRPL53*	39S ribosomal protein L53, mitochondrial	0.88
Q07021	*C1QBP*	Complement component 1 Q subcomponent-binding protein, mitochondrial	0.88
P60660	*MYL6*	Myosin light polypeptide 6	0.87
P82650	*MRPS22*	28S ribosomal protein S22, mitochondrial	0.87
Q14444	*CAPRIN1*	Caprin-1	0.87
Q99757	*TXN2*	Thioredoxin, mitochondrial	0.87
P21397	*MAOA*	Amine oxidase [flavin-containing] A	0.87
Q6NUK1	*SLC25A24*	Calcium-binding mitochondrial carrier protein SCaMC-1	0.87
P61812	*TGFB2*	Transforming growth factor beta-2 proprotein	0.86
Q13243	*SRSF5*	Serine/arginine-rich splicing factor 5	0.86
Q9UMY1	*NOL7*	Nucleolar protein 7	0.86
O14817	*TSPAN4*	Tetraspanin-4	0.86
Q9UII2	*ATP5IF1*	ATPase inhibitor, mitochondrial	0.86
Q10471	*GALNT2*	Polypeptide N-acetylgalactosaminyltransferase 2	0.86
Q92520	*FAM3C*	Protein FAM3C	0.86
Q13610	*PWP1*	Periodic tryptophan protein 1 homolog	0.85
P84090	*ERH*	Enhancer of rudimentary homolog	0.85
P67936	*TPM4*	Tropomyosin alpha-4 chain	0.85
Q14978	*NOLC1*	Nucleolar and coiled-body phosphoprotein 1	0.85
O14494	*PLPP1*	Phospholipid phosphatase 1	0.85
Q9NX58	*LYAR*	Cell growth-regulating nucleolar protein	0.85
Q9NY27	*PPP4R2*	Serine/threonine-protein phosphatase 4 regulatory subunit 2	0.85
Q5BKZ1	*ZNF326*	DBIRD complex subunit ZNF326	0.83
P20674	*COX5A*	Cytochrome c oxidase subunit 5A, mitochondrial	0.83
P09493	*TPM1*	Tropomyosin alpha-1 chain	0.83
P63098	*PPP3R1*	Calcineurin subunit B type 1	0.82
Q96EI5	*TCEAL4*	Transcription elongation factor A protein-like 4	0.82
Q9NPJ1	*MKKS*	McKusick-Kaufman/Bardet-Biedl syndromes putative chaperonin	0.82
P09497	*CLTB*	Clathrin light chain B	0.82
O15155	*BET1*	BET1 homolog	0.81
P63313	*TMSB10*	Thymosin beta-10	0.81
Q15293	*RCN1*	Reticulocalbin-1	0.81
Q15018	*ABRAXAS2*	BRISC complex subunit Abraxas 2	0.81
P10606	*COX5B*	Cytochrome c oxidase subunit 5B, mitochondrial	0.80
P07919	*UQCRH*	Cytochrome b-c1 complex subunit 6, mitochondrial	0.80
P49755	*TMED10*	Transmembrane emp24 domain-containing protein 10	0.80
Q99715	*COL12A1*	Collagen alpha-1	0.80
P05114	*HMGN1*	Non-histone chromosomal protein HMG-14	0.79
Q9P2N5	*RBM27*	RNA-binding protein 27	0.79

Q9H0A0	*NAT10*	RNA cytidine acetyltransferase	0.79
P05997	*COL5A2*	Collagen alpha-2	0.79
P60903	*S100A10*	Protein S100-A10	0.79
P30049	*ATP5F1D*	ATP synthase subunit delta, mitochondrial	0.78
P07602	*PSAP*	Prosaposin	0.77
P20962	*PTMS*	Parathymosin	0.76
P08123	*COL1A2*	Collagen alpha-2	0.75
P09496	*CLTA*	Clathrin light chain A	0.75
Q02818	*NUCB1*	Nucleobindin-1	0.75
P09486	*SPARC*	SPARC	0.75
P80723	*BASP1*	Brain acid soluble protein 1	0.74
P21127	*CDK11B*	Cyclin-dependent kinase 11B	0.73
P0DP25	*CALM3*	Calmodulin-3	0.72
Q9GZT3	*SLIRP*	SRA stem-loop-interacting RNA-binding protein, mitochondrial	0.71
P02461	*COL3A1*	collagen type III, alpha-1 chain	0.71
Q96A49	*SYAP1*	Synapse-associated protein 1	0.69
P20908	*COL5A1*	Collagen alpha-1(V)	0.67
Q15121	*PEA15*	Astrocytic phosphoprotein PEA-15	0.63
Q8N5N7	*MRPL50*	39S ribosomal protein L50, mitochondrial	0.62
P02452	*COL1A1*	Collagen alpha-1	0.53
